# Septic Arthritis Caused by Pasteurella multocida: A Source Control Issue

**DOI:** 10.7759/cureus.59537

**Published:** 2024-05-02

**Authors:** Beth Schwartz, Ashish Bhargava

**Affiliations:** 1 Internal Medicine, Ascension St. John Hospital, Detroit, USA

**Keywords:** septic arthritis, cat bite, hand infection, animal-related injuries, infectious arthritis, pasteurella multocida

## Abstract

*Pasteurella multocida* is known to be the most commonly isolated pathogen of soft tissue infections caused by cat or dog-inflicted wounds. We present a case of a 66-year-old female who was bitten by a cat, prescribed antibiotic therapy outpatient, and developed a septic metacarpophalangeal joint despite appropriate antibiotics. A failure to improve with appropriate antibiotic therapy should raise suspicion of a source control problem and prompt surgical intervention, a principle that is highlighted in this case. *Pasteurella multocida* septic arthritis of the fingers manifests in less than 4% of cases, making this case a rare presentation of a septic joint, which necessitated surgical management.

## Introduction

*Pasteurella multocida* is the most common cause of soft tissue infections related to cat or dog-inflicted wounds [1]. Only certain dog and cat bite wounds require prophylaxis, which includes high-risk injuries, such as those on the hand and those requiring surgical intervention [1]. We present a case of* Pasteurella multocida* joint infection that progressed despite outpatient antibiotic therapy. It is important for providers to be aware that, at times, patients do not improve on outpatient antibiotics because source control has not been achieved. Without this awareness, surgical intervention may be delayed in favor of changing antibiotic regimens.

## Case presentation

**Figure 1:**
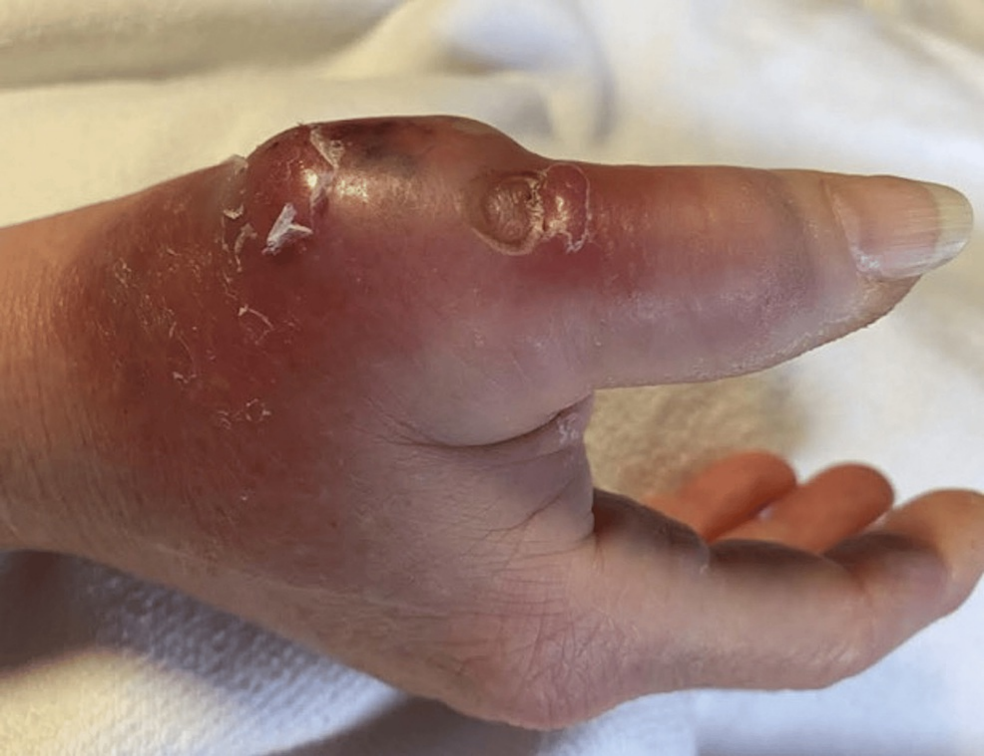
Photograph of the patient’s right thumb showing erythema and swelling

**Figure 2:**
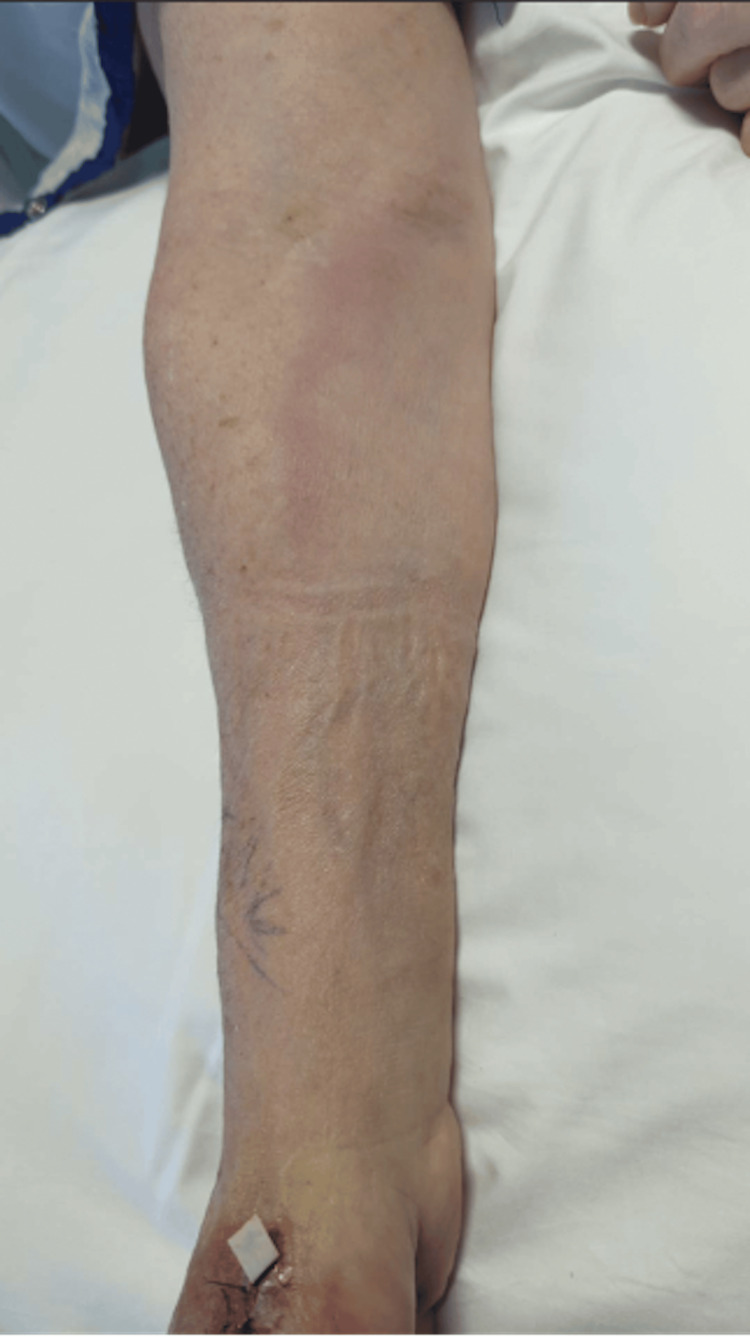
Photograph of the patient’s right arm showing lymphangitis

A 66-year-old female with type 2 diabetes mellitus presented with throbbing pain, erythema, and swelling of her right hand. She was bit by a feral cat approximately 20 days prior. Her hand became erythematous and painful a few days after the bite. She went to urgent care and was prescribed amoxicillin-clavulanate for 10 days. She completed this course; however, the erythema and pain worsened. She denied nausea, vomiting, or abdominal pain. The patient's vitals were blood pressure of 120/70, respiratory rate of 16, heart rate of 88, and temperature of 97.1°F at the clinic. Examination showed tenderness to palpation of the right thumb with swelling and erythema (Figure 1). Labs included a white blood cell count of 9.60 K/mcL, creatinine at 0.61 mg/dL, and glucose at 116 mg/dL (Table 1). X-rays of the right hand were negative for soft tissue gas or bony destruction. This patient was admitted and started on ampicillin-sulbactam. Hand surgery was performed for incision and drainage with arthrotomy and metacarpophalangeal joint washout. The next day, new fluctuance of the volar aspect, lymphangitis extending up the right arm, and axillary lymphadenopathy were noted (Figure 2). She returned to surgery and a deep thenar abscess was incised and drained, with washout of the thumb flexor sheath and debridement of necrotic skin. Wound cultures grew *Pasteurella multocida* sensitive to penicillin. She improved and was discharged on amoxicillin-clavulanate to complete four weeks of antibiotics. She was seen in the clinic in follow-up and was doing well, with complete symptom resolution, three months later.

**Table 1:** Laboratory values

Laboratory tests (units)	Result	Reference range
Hemoglobin (gm/dL)	11.0	12.0-16.0
White blood cells (K/mcL)	9.60	4.00-11.00
Platelets (K/mcL)	405	150-400
Creatinine (mg/dL)	0.61	0.70-1.20
Glucose, random (mg/dL)	116	70-200
Sodium (mmol/L)	141	135-145
Potassium (mmol/L)	4.5	3.5-5.0
Chloride (mmol/L)	105	98-109
Bicarbonate (mmol/L)	23	23-34
Anion gap (mmol/L)	13	4-14

## Discussion

*Pasteurella multocida,* a gram-negative coccobacillus, is the most common etiology of soft tissue infections resulting from cat (and dog) bites or scratches [1]. Approximately 70-90% of cats carry *Pasteurella* species. *Pasteurella *can be isolated in up to 75% of cultures from cat bites [2]. Infection usually occurs within 24 hours of the inciting wound with erythema, pain, and purulence at the site. If cellulitis occurs, this is typically evident within two days [3]. In a study by Talan et al., 28% of cat bite patients developed lymphangitis, and 63% of those cases were caused by *Pasteurella *species [2].

Prophylaxis is only recommended for those with comorbidities conferring immunosuppression, underlying joint degeneration or replacement, and high-risk wounds [3]. High-risk wounds include those on the face, hand, or genitals, as well as deep wounds and those requiring surgical intervention [1,4]. Prophylaxis is typically done with a five-day course of oral amoxicillin-clavulanate [5]. A 25-30% mortality rate of *Pasteurella* infections has been reported [6]. Pasteurellosis can progress to bacteremia, meningitis, osteomyelitis, and endocarditis [7]. If complications such as systemic signs of infection or spread to deeper tissues occur, intravenous antibiotics are administered. Surgical intervention is recommended for those with deeper infections, abscesses, or necrotizing infection [5]. Previous studies have estimated that septic arthritis caused by* Pasteurella multocida* manifests in the fingers, as in this case, in only 3.8% of cases [7]. First-line intravenous antibiotic therapy is typically ampicillin-sulbactam [2]. A retrospective study by Giordano et al. found that 48% of patients with pasteurellosis infection required surgical intervention; however, data are limited as to how many pasteurellosis cases require repeat intervention as in this case [1].

## Conclusions

This case highlights the importance of close follow-up for high-risk patients suffering from bite wounds. When outpatient antibiotics fail, a source control issue should be suspected and prompt urgent evaluation. Septic arthritis may be complicated by a lack of source control, requiring repeat surgical interventions, as in this case of *Pasteurella* infection. A high suspicion index is necessary to promptly care for patients with deeper, complicated infections.
